# Correlates of objective sleep quality in older peritoneal dialysis patients

**DOI:** 10.1080/0886022X.2020.1871369

**Published:** 2021-01-17

**Authors:** Haifen Zhang, Yan Yang, Jiaying Huang, Shuhui Lailan, Xingjuan Tao

**Affiliations:** a Department of Nephrology, Renji Hospital, School of Medicine, Shanghai Jiao Tong University, Shanghai, China; b Nursing Department, Renji Hospital, School of Medicine, Shanghai Jiao Tong University, Shanghai, China; c School of Nursing, Shanghai Jiao Tong University, Shanghai, China

**Keywords:** Sleep disturbance, dialysis, kidney disease, actigraphy, sleep monitoring

## Abstract

**Background:**

Sleep disturbance is a prominent concern in dialysis patients and detrimentally impacts clinical and self-reported health outcomes. This study aimed to collect sleep data from in-home actigraphy and to explore possible predictors of sleep quality in older peritoneal dialysis patients.

**Methods:**

This was a cross-sectional study. Peritoneal dialysis patients aged ≥60 years participated in this study. For each participant, sleep quality was assessed by analyzing the data produced by an actigraphic device worn on the wrist 24 h a day for seven consecutive days. Physical function was assessed using handgrip strength and the Timed Up and Go test. Depression was assessed using the self-reported Geriatric Depression Scale. Multiple linear regression analyses were performed to examine the factors influencing sleep efficiency and sleep time.

**Results:**

Based on data collected from 50 participants (*N* = 50, mean age 70.4 years, 70% male), including 333 nights of actigraphy-monitored sleep, the mean sleep efficiency was 75.5%±14.2% and the mean total sleep time 391.0 ± 99.3 min per night. Higher hemoglobin (β = 0.38, *p* = 0.007) and lower serum phosphorus (β = −0.30, *p* = 0.042) levels were significant predictors of better sleep efficiency. The only significant predictor of the total sleep time was age (β = 0.32, *p* = 0.021).

**Conclusion:**

Older peritoneal dialysis patients had poor sleep, characterized by low sleep efficiency. Low hemoglobin and high serum phosphorus levels were predictors of poor sleep efficiency and, as such, modifiable factors for clinicians to consider when treating patients with sleep complaints.

## Introduction

Sleep disturbance, including difficulty falling and staying asleep at night, is a prominent concern of dialysis patients and their caregivers [1]. Poor sleep is not only associated with frailty, cognitive impairment, and decreased quality of life, but also increases the risk of cardiovascular complications and mortality in adult dialysis patients with chronic kidney disease [2–5]. Sleep disturbance is highly prevalent among end-stage renal disease patients undergoing dialysis treatment, ranging from 39% to 80% [6–8]; this broad range of prevalence may be attributable partly to differences in sleep measures and study populations. During the last decade, the incidence of end-stage renal disease in elderly patients has also been steadily increasing worldwide [9]. Data from the European register in 2017 showed that 52% of patients initiating renal replacement therapy were aged ≥ 65 years [10]. In China, the mean age of dialysis patients with chronic kidney disease in 2015 was 55 years [11]. Aging is associated with poor quality of sleep, decreased total sleep time, reduced sleep efficiency, more frequent awakenings, and prolonged nocturnal awakening [12]. Unsurprisingly, poor sleep quality also closely correlates with older age among adult dialysis patients in general [13].

Older adults tend to have multiple comorbidities and complications. Prior research documented that a large proportion of sleep complaints could be attributed to older adults’ poor health conditions and emotional distress [14]. Poor sleep has been, additionally, associated with a variety of disease and treatment-specific factors such as uremia, residual renal function, increased levels of inflammatory cytokines, anemia, and phosphorus in renal patients [15–17]. Both functional and psychological factors, as well as those related to renal disease pathology and secondary to the effects of chronic dialysis, probably play significant roles in sleep disruption among older adults on dialysis. However, sleep disturbances among older adults on dialysis have received little attention. In addition, many older adults are less likely to report sleep problems because they regard such problems as a normal part of aging [12]. Given that actigraphy can reliably measure sleep time, sleep efficiency, and sleep-wake rhythms, the aim of our study was to collect sleep data using actigraphy and to explore the possible predictors of sleep quality in older peritoneal dialysis patients. We hypothesize that older peritoneal dialysis patients experience impaired sleep quality and that both geriatric and disease-specific factors significantly correlate with objectively measured sleep duration and/or quality.

## Methods

### Study design

This cross-sectional, descriptive study involved a convenience sample of patients recruited from an outpatient peritoneal dialysis clinic at a university-affiliated hospital in Shanghai, China, between August 2018 and November 2019. We included patients who were aged ≥60 years with end-stage renal disease undergoing peritoneal dialysis treatment for >3 months, able to walk without assistance, and able to understand and complete the interviewer-administered questionnaires. The exclusion criteria were as follows: mental illness, conditions causing physical instability; and hospitalization or records of sleep medication prescriptions during the preceding 3 months. G* Power was used to estimate the sample size. Data from the first 40 participants were used to calculate the study design power [18]. The effect size for the total sleep time was 0.26. Based on multiple linear regression, a total sample size of 49 was needed to achieve 80% power with a 5% significant level. Therefore, the sample size was set at 50. The study was approved by the ethics committee of the affiliated university (SJUPN-201705). Prior to data collection, written informed consent was obtained from each participant. The funding source of the current study had no conflicts of interest in the data collection, analyses, and interpretation, or manuscript preparation.

### Sleep monitoring

To measure the sleep quantity and quality, all study participants wore an actigraph (an accelerometer-based motion-detecting portable device) on the non-dominant wrist 24 h a day for seven consecutive days (ActiGraph GT3X, Pensacola, FL, USA). An actigraph is a compact, wristwatch-like, noninvasive triaxial accelerometer that can be used to monitor human rest/activity cycles. The actigraphic device was initialized using 60-s epochs at a frequency of 30 Hz. It was then worn on the participant’s wrist to continually record gross motor activity. Wrist actigraphy can accurately identify individuals with poor sleep quality by comparing with the results of polysomnography in patients with end-stage renal disease [19]. The participants were also instructed to record the times at which they went to bed and woke up in the morning during the actigraph recording period. Upon return of the device, we downloaded and analyzed the data using ActiLife V6.13.3 software (ActiGraph, Pensacola, FL, USA). The recorded sleep times were used to detect the sleep period during the data analysis process. If sleep period information was missing or appeared to be invalid, we used the ActiLife-defined sleep interval based on the activity pattern. Wear time was validated using the Choi algorithm [20], where 90-min consecutive zero counts with a 2-min spike tolerance were screened as nonwear if zero counts were detected during both the 30-min upstream and downstream from that interval. Participants were excluded from our study if the actigraphic data showed <4 days of wear time. Sleep parameters, including total sleep time (total number of sleep minutes during the main rest interval) and sleep efficiency (total number of sleep minutes divided by the number of minutes in bed), were determined through the Cole-Kripke algorithm [21]. The algorithm determines if each epoch corresponds to an awake or sleep state based on a 7-min window including the four previous epochs and two epochs after the current one. The epoch is considered “asleep” if the result of the algorithm is <1.

### Testing of physical function, depression symptoms, and cognition

We assessed the physical function, depression symptoms, and cognition of each participant on the day of actigraphy data collection. To assess the patients’ physical function, we performed the following two tests: first, a handgrip strength test using a hand dynamometer with the participant in a seated position with the elbow in 90° flexion and the wrist in neutral position. Participants were instructed to apply maximum grip strength three times with the dominant hand, and the highest grip strength was scored for each participant. Second, we used the Timed Up and Go test, which measures the time it takes for the patient to stand up from a chair, walk 3 m, and return to the seated position.

Depressive symptoms were evaluated using the self-reported Geriatric Depression Scale (GDS), which consists of 15 dichotomous questions. The possible scores range from 0 to 15, with a higher score indicating more depressive symptoms. The Chinese version of GDS has a Cronbach’s alpha of 0.774 and is considered reliable [22]. A score of ≥5 is reported to have high specificity (95%) and sensitivity (97%) for the identification of major depressive disorders in older Asian adults [23]. In this study, Cronbach’s alpha for the GDS was 0.709.

The Mini-Mental State Examination was used to assess the participants’ global cognition. The test can be scored between 0 and 30, with a higher score indicating better performance. This test has acceptable sensitivity (87.6–94.3%) and specificity (80.0–94.3%) for detecting dementia in the older population [24]. In this sample, Cronbach’s alpha for this test was 0.656.

### Physical activity questionnaire

Physical activity was assessed using the International Physical Activity Questionnaire (IPAQ)-short version. The questionnaire was developed by an international consensus group in 1998. The IPAQ has two versions: the long version and the short form (IPAQ-SF), which collects information on the amount of time spent in three types of physical activity (vigorous, moderate, and walking) during the preceding 7 days, as well as the amount of sedentary time during the same period. The total physical activity score derived from this questionnaire substantially correlates with the pedometer-measured activity (*r* = 0.561) in the dialysis patient population, indicating that the questionnaire has satisfactory validity [25]. The IPAQ-SF score was converted into metabolic equivalent minutes per week (MET-min/week) based on the scoring manual [26].

### Demographic and clinical questionnaire

A demographic and clinical questionnaire was developed by the researchers. It included demographic information such as age, sex, marital status, educational level, monthly income, and living status as well as clinical information such as dialysis duration, the main cause of end-stage renal disease, height, weight, and biochemical information. The blood sample for the biochemical evaluation was collected on the day of questionnaire data collection, after overnight fasting. It included estimated glomerular filtration rate, creatinine, urea, serum albumin, prealbumin, hemoglobin, phosphate, serum calcium, intact parathyroid hormone (iPTH), and potassium. Hemoglobin and serum phosphorus levels were determined using colorimetry, and iPTH levels were measured using chemoluminescence.

### Data collection

All questionnaire data were collected through face-to-face interviews by a trained nurse at the peritoneal dialysis outpatient clinic. Demographic and clinic information were retrieved from the patients’ medical charts. Physical performance tests were conducted by the same trained nurse using standardized protocols.

### Statistical analyses

SPSS® version 21.0 (IBM Corporation, Armonk NY, USA) for Windows® was used for the statistical analysis. The participants’ demographic and clinical characteristics were analyzed using means (standard deviation) or frequencies (proportions). The assumption of normality was evaluated using the Shapiro–Wilk normality test. The independent sample *t*-test or analysis of variance was adopted to identify potential influencing factors for sleep efficiency and total sleep time as appropriate. Pearson’s or Spearman’s tests were conducted to determine the associations between GDS scores, physical function, cognition, and sleep quality. For univariable prescreening of candidate covariates, a *p*-value of 0.05 is only recommended for very large sample sizes (events per variable ≥100), while a value of 0.2 or even 0.5 was recommended to avoid important variables being missed from a multiple regression model owing to stochastic variability [27]. Variables with *p* < 0.2 based on bivariate analyses, or those described as relevant in the literature, were included in our multiple linear regression analyses of the factors influencing sleep efficiency and total sleep time. Our regression models were adjusted for age, sex, and body mass index. The variance inflation factor (VIF) (<10) and Tolerance (>0.1) were checked to avoid multicollinearity problems for each independent variable. The homoscedasticity (normal distribution of residuals) was checked by inspecting the standardized residual using predicted plots. Durban–Watson’s d tests were used to examine autocorrelation in the data with d values 0–4, indicating no autocorrelation. All tests were two-tailed, and *p* < 0.05 was considered significant.

## Results

### Participant characteristics

Fifty-six patients participated in the 7-day actigraphy assessment, but six patients did not have adequate wear time. Only the data from 50 patients were therefore included in our analyses. The patients’ mean age was 70.4 years (interquartile range, 64.0–76.0 years), and 70.0% were male. The mean dialysis vintage was 62.5 months (interquartile range, 35.0–76.3 months). Chronic glomerulonephritis (30.0%) was the main cause of end-stage renal disease in our sample. The demographic and clinical characteristics of all study participants are shown in Table 1.

**Table 1.** Demographic and clinical characteristics of all study participants.

Characteristics	Total sample (*N* = 50)
	Mean ± SD or *N* (%)
Age	70.4 ± 7.2
Sex	
Women	15 (30.0)
Men	35 (70.0)
Marital status	
Single or widowed	6 (12.0)
Married	43 (86.0)
Missing data	1 (2.0)
Educational level	
Junior high school or below	30 (60.0)
Senior high school or above	20 (40.0)
Residence	
Downtown	12 (24.0)
Suburban	38 (76.0)
Living status	
With spouse	31 (62.0)
With others or alone	19 (38.0)
Monthly income	
<3000 RMB	11 (22.0)
≥3000 RMB	39 (78.0)
Body mass index (kg/m^2^)	24.1 ± 2.8
Primary renal disease	
Chronic glomerulonephritis	15 (30.0)
Diabetic nephropathy	7 (14.0)
Hypertensive nephrosclerosis	1 (2.0)
Polycystic kidney disease	4 (8.0)
Unknown	16 (32.0)
Other	7 (14.0)
Dialysis vintage (months)	62.5 ± 42.7
Peritoneal dialysis type	
Self-care peritoneal dialysis	41 (82.0)
Assisted peritoneal dialysis	9 (12.0)
Number of prescribed medications	10.9 ± 5.1
Estimated glomerular filtration rate (mL/min/1.73 m^2^)	2.1 ± 2.1
Serum creatinine (µmol/L)	799.1 ± 303.7
Serum urea (mmol/L)	29.8 ± 25.3
Weekly KT/V (renal + peritoneal)	1.9 ± 0.4
Normalized protein catabolic rate (g/kg/day)	0.8 ± 0.2
Serum albumin (g/L)	36.2 ± 3.6
Serum pre-albumin (mg/L)	328.2 ± 59.4
Hemoglobin (g/L)	112.5 ± 17.1
Phosphate (mmol/L)	1.5 ± 0.3
Serum calcium (mmol/L)	2.4 ± 1.1
Intact parathyroid hormone (ng/L)	465.3 ± 407.8

SD: standard deviation; Kt/V represents the dose of hemodialysis, an abbreviation of (K_urea_×T_d_)/V_urea_. K_urea_ (milliliters/minute).

### Actigraphy results, health condition, and physical activity of the participants

Our study participants produced 333 nights of actigraphy data. Based on our analysis of these data, the mean sleep efficiency was 75.5%±14.2% and the mean total sleep time was 391.0 ± 99.3 min. The mean handgrip strength and GDS scores were 26.3 ± 8.1 kg and 6.7 ± 3.1, respectively. The mean physical activity level score was 1040.9 ± 973.2 MET-min/week, and walking was the major component of MET energy expenditure (Table 2).

**Table 2.** Sleep quality, physical function, depression symptoms, cognition, and physical activity of study participants.

Variables	Mean ± SD, *n* (%)
Total sleep time (minutes)	391.0 ± 99.3 (6.5 ± 1.7 hours)
Sleep efficiency (%)	75.5 ± 14.2
Handgrip strength (kg)	26.3 ± 8.1
Male	29.0 ± 7.5
Female	19.9 ± 5.6
TUG (s)	10.9 ± 4.3
MMSE score	27.2 ± 2.3
GDS	6.7 ± 3.1
IPAQ (MET·min·wk^−1^)	
Total activity	1040.9 ± 973.2
Vigorous activity	0
Moderate activity	210.1 ± 643.1
Walking	830.9 ± 645.4

TUG: Timed Up and Go; MMSE: Mini-Mental State Examination; GDS: Geriatric Depression Scale; IPAQ: International Physical Activity Questionnaire; SD: standard deviation; MET: Metabolic Equivalent Task.

### Bivariate correlations among study variables

Table 3 shows the results of the statistical analysis of the sleep efficiency and total sleep time of study participants according to their characteristics and Table 4 the correlations among sleep efficiency; total sleep time; and participants’ clinical, physical, and mental status. A higher sleep efficiency was associated with an older age (*r* = 0.30, *p* < 0.05) and better hemoglobin levels (*r* = 0.36, *p* < 0.05) and correlated with lower serum phosphorus levels (*r* = −0.36, *p* < 0.01). A longer total sleep time was associated with an older age (*r* = 0.41, *p* < 0.01) and correlated with lower serum phosphorus levels (*r* = −0.39, *p* < 0.01).

**Table 3.** Statistical analysis of sleep efficiency and total sleep time with respect to patients’ characteristics.

Variables	Sleep efficiency	Total sleep time
	(Mean ± SD)	(Mean ± SD)
Sex		
Female	77.9 ± 12.0	402.5 ± 84.6
Male	74.5 ± 15.1	386.1 ± 105.8
*P*-value	0.455	0.598
Educational level		
Junior high school or below	76.1 ± 13.4	400.9 ± 89.4
Senior high school or above	74.7 ± 15.7	376.2 ± 113.4
* P*-value	0.803	0.395
Marital status		
Single	74.9 ± 12.1	394.3 ± 150.3
Married	76.3 ± 13.9	395.4 ± 88.2
* P*-value	0.719	0.980
Residence		
Downtown	76.9 ± 11.5	405.5 ± 89.2
Suburban	71.1 ± 20.6	345.2 ± 119.0
* P*-value	0.338	0.066
Living status		
With spouse	73.3 ± 13.96	373.3 ± 86.9
With others or alone	79.1 ± 14.3	419.9 ± 113.3
*P*-value	0.101	0.108
Monthly income		
<3000 Chinese Yuan	73.8 ± 18.2	372.0 ± 104.0
≥3000 Chinese Yuan	76.0 ± 13.1	396.4 ± 98.7
*P*-value	0.762	0.479
Peritoneal dialysis type		
Self-care peritoneal dialysis	76.3 ± 13.0	390.0 ± 92.2
Assisted peritoneal dialysis	72.1 ± 19.1	395.8 ± 133.9
*P*-value	0.517	0.876
Diabetes		
Yes	75.0 ± 14.8	382.5 ± 94.6
No	75.8 ± 14.1	395.8 ± 103.1
*P*-value	0.847	0.656

SD: standard deviation.

**Table 4.** Bivariate correlations between independent and dependent variables.

	Dependent variables	
Independent variables	SE	TST
Age (years)	0.295*	0.407**
Weekly Kt/V	0.204	0.200
Hemoglobin (g/L)	0.360*	0.239
Serum albumin (g/L)	0.143	0.173
Phosphorus (mmol/L)	−0.363**	−0.390**
Handgrip strength (Kg)	−0.065	−0.088
TUG (s)	0.148	0.176
GDS	0.193	0.166

TUG: Timed Up and Go; GDS: Geriatric Depression Scale; SE: sleep efficiency; TST: total sleep time; Kt/V represents the dose of hemodialysis, an abbreviation of (K_urea_×T_d_)/V_urea_. K_urea_ (milliliters/minute).

* *p <* 0.05, ** *p <* 0.01.

### Linear regression models predicting sleep efficiency and total sleep time

Table 5 presents the multiple linear regression models for the predictors of sleep efficiency and total sleep time. VFIs and tolerance were within acceptable ranges: the correlation coefficients among independent variables < 0.7 and Durban–Watson’s d values between 0 and 4. Hemoglobin (β = 0.38) and serum phosphorus (β = −0.30) levels were significant predictors of sleep efficiency. The proportion of variance in sleep efficiency explained by these variables was 34.8%. Only age (β = 0.32) significantly predicted the total sleep time, and 38.4% of the variances in total sleep time was explained by that factor. Similar results were obtained after adjustment for GDS scores and physical activity (Table 6).

**Table 5.** Linear regression analyses of sleep efficiency and total sleep time.

Sleep quality and variables	*SE* β (95% CI)	*p* Value
Sleep efficiency		
Age	0.241 (−0.059–1.101)	0.080
Female sex (reference: male)	0.205 (−2.292–14.864)	0.147
Body mass index	0.037 (−1.183–1.562)	0.782
Living status	0.126 (−4.118–11.415)	0.349
Weekly Kt/V	−0.079 (−12.776–7.416)	0.595
Hemoglobin^a^	0.377 (0.091–0.534)	0.007
Phosphorus^a^	−0.300 (−28.088 to −0.540)	0.042
* R^2^*	0.348	
Total sleep time		
Age^a^	0.318 (0.012–0.134)	0.021
Female sex (reference: male)	0.144 (−0.467–1.494)	0.296
Body mass index	−0.057 (−0.191–0.124)	0.667
Living status	0.131(−0.448–1.335)	0.321
Residence	−0.188 (−1.686–0.243)	0.139
Weekly Kt/V	−0.011 (−1.200–1.115)	0.941
Hemoglobin	0.210 (−0.005–0.046)	0.118
Phosphorus	−0.273 (−3.091–0.068)	0.060
* R^2^*	0.384	

*SE* β: standardized coefficient; CI: confidence interval; Kt/V represents the dose of hemodialysis, an abbreviation of (K_urea_×T_d_)/V_urea_. K_urea_ (milliliters/minute).

^a^Significant predictors (*p <* 0.05).

**Table 6.** Linear regression analyses of sleep efficiency and total sleep time (adjusted for GDS score and physical activity).

Sleep quality and variables	*SE* β (95% CI)	*p* Value
Sleep efficiency		
Age	0.252 (−0.056–1.051)	0.077
Female sex (reference: male)	0.181 (−3.324–14.470)	0.213
Body mass index	0.013 (−1.365–1.493)	0.928
Living status	0.102 (−5.218–11.126)	0.469
Weekly Kt/V	−0.043 (−12.117–9.233)	0.786
GDS score	0.122 (−0.792–1.919)	0.406
Physical activity	0.051 (−0.003–0.005)	0.711
Hemoglobin^a^	0.351 (0.061–0.522)	0.015
Phosphorus^a^	−0.303 (−28.462 to −0.423)	0.044
* R^2^*	0.360	
Total sleep time		
Age^a^	0.315 (0.011–0.133)	0.021
Female sex (reference: male)	0.112 (−0.639–1.443)	0.440
Body mass index	−0.113 (−0.228–0.094)	0.407
Living status	0.069 (−0.685–1.148)	0.612
Residence	−0.233 (−1.869–0.082)	0.071
Weekly Kt/V	0.038 (−1.080–1.382)	0.806
GDS score	0.227 (−0.030–0.273)	0.123
Physical activity	−0.047 (−1.139–0.861)	0.740
Hemoglobin	0.179 (−0.011–0.045)	0.221
Phosphorus	−0.265 (−3.049– 0.109)	0.067
* R^2^*	0.428	

*SE* β: standardized coefficient; CI: confidence interval; Kt/V represents the dose of hemodialysis, an abbreviation of (K_urea_×T_d_)/V_urea_. K_urea_ (milliliters/minute); GDS, Geriatric Depression Scale.

^a^Significant predictors (*p <* 0.05).

## Discussion

The present study demonstrated that poor sleep quality was a reality among elderly peritoneal dialysis patients, while the results of regression analyses supported the hypothesis that disease-specific factors such as higher hemoglobin and lower serum phosphorus levels were associated with longer total sleep duration and better sleep efficiency; however, geriatric factors such as handgrip strength and depressive symptoms were not found to be associated with sleep quality parameters. These findings suggest that, in older peritoneal dialysis patients, better anemia control and optimizing the management of mineral and bone disorders may improve the patients’ sleep quality.

Using actigraphy data, this study confirms that a higher number of older peritoneal dialysis patients experience disturbed sleep than older adults in general. The average sleep efficiency in our study participants (75.5%±14.2%) was worse than that of the healthy older adult population reported in Alfini et al.’s study [28]. They found a mean actigraphy measured sleep efficiency of 83.4%±7.6% for 392 cognitively normal community-dwelling older adults with a mean age of 73.1 years old. Compared with that of conventional adult hemodialysis patients, the sleep efficiency of older peritoneal dialysis patients is also lower (84.8%±7.1% versus 75.5% ± 14.2%) [29].

Even though the average sleep efficiency among elderly peritoneal dialysis patients was suboptimal, a positive association between age and sleep efficiency was observed in the present study. Additionally, older peritoneal dialysis patients had higher average total sleep time (391.0 ± 99.3 min) than the general adult dialysis population, as reported previously, wherein the average total sleep time (on actigraphy) was 349.9 ± 63.9 min among conventional hemodialysis patients with a mean age of 47.6 years [29], and 5.5 ± 1.3 h among peritoneal dialysis patients with a mean age of 60.2 years [30]. Furthermore, age was found to be a positive predictor of the total sleep time in this study. This finding is contrary to the general understanding that even healthy adults experience a decrease in sleep duration and efficiency as they age [31]. Previous studies of hemodialysis patients have also reported that age is negatively associated with both subjective and objective sleep quality [7,13]. This inconsistency may be explained by the differences in study samples, as the literature involves a wide range of age groups, including but not limited to the older population. Among healthy participants aged ≥60 years, a meta-analysis review revealed that the negative relationship between sleep duration and age was no longer significant [32]. A recent longitudinal study revealed that the older group had higher sleep problems at outset than the younger group, but a steep increase in sleep time and fewer sleep problems over time were observed in the older group [33]. In China, compared with the older elderly, the younger elderly are more likely to take on grandchild/spouse care and household responsibilities, which may negatively impact their sleep quality. Different age groups may possibly have different trajectories related to sleep disturbance. It would be interesting to further determine sleep problems among various age groups of elderly dialysis patients considering various social contexts.

In our study, low hemoglobin levels were significantly correlated with low levels of sleep efficiency. Indeed, the hemoglobin levels appear to be related to sleep quality in the general older adult population as well as in the dialysis patient population; the English Longitudinal Study of Aging (involving 6465 participants aged 50–99 years) revealed that sleep disturbance was related to low hemoglobin levels and anemia [34]. Consistently, it has been reported that adult dialysis patients with hemoglobin levels between 10 and 12 g/dL had better subjective sleep efficiency than those with lower hemoglobin levels [35]. It is worth noting that hemoglobin levels decrease with age in the general population owing to the changes in sex hormone secretion and erythropoiesis in the bone marrow. Previous observational studies in hemodialysis patients revealed that older patients had significantly lower hemoglobin levels than nonolder patients [36,37]. Given that iron is bound to hemoglobin, a decrease in hemoglobin levels may cause a decrease in iron levels. Thus, anemia is a common condition in dialysis patients. Brain iron deficiency may also be involved in the pathogenesis of restless legs syndrome [38], a common health problem in the dialysis population that is often related to sleep fragmentation [39,40]. Alternatively, older peritoneal dialysis patients may produce less erythropoietin owing to altered renal function, and this may reduce the levels of iron transported to the medulla and central nervous system, which may further impair the efficiency of sleep.

In the current study, high serum phosphorus levels were associated with poor sleep efficiency in older peritoneal dialysis patients—a result consistent with a previous international study in which a steady rise in the odds of having poor sleep quality was found in patients with higher levels of serum phosphorus [4]. This relationship between self-reported sleep quality and pre-dialysis phosphorus was also noted by Unruh et al. [41]. In patients with chronic kidney diseases, a high serum phosphorus level is a major cause of pruritus, which may subsequently cause sleep disturbance. While many studies have failed to identify a significant relationship between serum phosphorus levels and pruritus [42,43], high levels of phosphorus may impair sleep in other ways. Phosphate and vitamin D metabolisms are reported to be interconnected and related to the activity of klotho, the aging-regulating gene [44,45]. Phosphate load may lead to increased fibroblast growth factor 23, decreased vitamin D, increased intact parathyroid hormone, and decreased klotho; such a vicious cycle is likely activated even before hyperphosphatemia occurs [46]. A previous cross-sectional study involving 141 hemodialysis patients revealed a significant association between low levels of vitamin D and poor sleep quality [47]. It may be that a decrease in phosphorus-inducing vitamin D further deteriorates age-related vitamin D deficiency, which negatively impacts sleep efficiency in older peritoneal dialysis patients.

Our study is first limited by the small sample size and the multiplicity of variables in our statistical analysis. Second, it is possible that the sleep diaries of the participants or their caregivers did not accurately record bedtimes and awakenings. Third, we did not ask the participants to report their subjective sense of sleep quality; thus, these data were not available. Fourth, actigraphy may overestimate sleep owing to its limitation in differentiating quiet wakefulness from sleep. A comprehensive assessment, including objectively measured sleep efficiency, the patient’s subjective sense of sleep quality, and the patient’s subjective sense of the impact of sleep quality on daytime function (i.e., fatigue, headaches) may further improve our understanding of sleep quality among older peritoneal dialysis patients. Fifth, we did not control for certain medications that may influence sleep quality, such as beta-blockers and statins as well as alcohol, tea, and coffee consumption. Finally, our study was cross-sectional in design and, therefore, cannot firmly establish the causal nature of the studied variables.

In conclusion, our study indicated that older peritoneal dialysis patients are likely to have suboptimal sleep quality. Within our sample, low hemoglobin and high serum phosphorus levels (in addition to age) were the primary predictors of poor sleep efficiency. Significantly, these are treatable factors for clinicians to consider when assisting patients who complain of disturbed sleep. Given that older adults are a fast-growing subset of the peritoneal dialysis population, further research examining specific interventions aimed at balancing phosphate and vitamin D levels, or better anemia control, may be enormously beneficial.
